# The Association of HOTAIR with the Diagnosis and Prognosis of Gastric Cancer and Its Effect on the Proliferation of Gastric Cancer Cells

**DOI:** 10.1155/2019/3076345

**Published:** 2019-06-09

**Authors:** Zhiying Xu, Hui Chen, Bin Yang, Xiangfeng Liu, Xiaoli Zhou, Hongfang Kong

**Affiliations:** ^1^Department of Gastroenterology, People's Hospital of Taizhou, 366 Taihu Road, Taizhou 225300, Jiangsu, China; ^2^Department of General Surgery, 2nd Xiangya Hospital, Central South University, 139 Renmin Middle Road, Changsha, Hunan 410011, China

## Abstract

**Background:**

Long noncoding RNAs (lncRNAs) are a group of noncoding RNA with the length of more than 200nt. They have been identified as important diagnostic and prognostic molecules for many cancers and play an important role in the development of cancers. However, their clinical value and roles in gastric cancer (GC) remain unclear.

**Methods:**

The expression levels of HOTAIR in 54 GC tissues and their matched adjacent nontumor tissues from GC patients and 24 normal mucosa or those with minimal gastritis as healthy controls were determined by qRT-PCR. The expression levels of HOTAIR in human GC cell lines and a normal gastric epithelium cell line were also assessed by qRT-PCR. The potential relationships between its level in GC tissues and the clinicopathological features were analyzed. Furthermore, a receiver operating characteristic (ROC) curve was constructed. Additionally, the correlation between this lncRNA and overall survival (OS) was analyzed. SiRNA transfection was used to silence the expression of HOTAIR in GC cells. And cell proliferation and cell cycle assays were employed to determine the effect of HOTAIR on GC cell growth. Western blot was performed for the detection of the P53, P21, and Bcl2 proteins.

**Results:**

The expression levels of HOTAIR were significantly upregulated in GC tissues and cell lines. Increased HOTAIR was associated with tumor differentiation, lymph node and distant metastasis, and clinical stage. Furthermore, the area under the ROC curve (AUC) was up to 0.8416 (95 % CI=0.7661 to 0.9170, P<0.0001). The sensitivity and specificity were 66.67 and 87.04%, respectively. The correlation between HOTAIR expression and overall survival (OS) was statistically significant. The hazard ratio was 2.681, and 95% CI of ratio was 1.370 to 5.248. In addition, knockdown of HOTAIR can inhibit GC cell growth and affect cell cycle distribution. And knockdown of HOTAIR could enhance the protein levels of P21 and P53.

**Conclusion:**

The present study demonstrated that HOTAIR was highly expressed in GC tissues and may serve as a potential diagnostic and prognostic biomarker for GC. And HOTAIR promoted GC cell proliferation.

## 1. Introduction

Gastric cancer (GC) is one of the most common malignant diseases and the third leading cause of cancer deaths in the world. Despite the great advancement in diagnosis and treatment for GC, patients with GC still have poor prognosis due to tumor recurrence and metastasis [1]. Therefore, there is an urgent need to better understand the molecular pathogenesis of GC in order to identify new biomarkers and targets for effective therapy. GC is a consequence of multifactors, including environmental and genetic factors. However, the molecular and cellular mechanisms of GC pathogenesis remain unclear.

Long noncoding RNAs (lncRNAs) are a novel class of noncoding RNA with the length >200nt but no coding capacity. In the last few decades, lncRNAs had been considered as ‘‘transcriptional noise.” Nonetheless, accumulating evidence has suggested that lncRNAs are participated in many cellular processes, such as stem cell pluripotency, cell growth and apoptosis, and human disease pathogenesis [2–5]. The disorders of lncRNA play a critical role in the initiation and development of cancers. lncRNAs can regulate cancer cell growth and apoptosis as well as cancer progression and metastasis. And they might be suitable as potential diagnostic biomarkers and therapeutic targets for cancers [6–10]. A growing number of studies have shown that several lncRNAs are involved in the pathogenesis of GC [11–13]. The lncRNA HOXA cluster antisense RNA2 (HOXA-AS2) was upregulated in GC, and upregulated HOXA-AS2 could promote gastric cancer proliferation [14]; lnc01614 was significantly higher in GC tissues, and it promotes the occurrence and development of GC [15]; a pseudogene-derived lncRNA SFTA1P is significantly downregulated in GC tissues, and SFTA1P can suppress cell proliferation, migration, and invasion in gastric cancer [16]. Additionally, the lncRNAs NEAT1, UCA1, CASC15, and so on have been demonstrated to be implicated in GC [17–22].

In this study, we showed that HOTAIR is upregulated in GC tissues and cells. HOTAIR could promote GC cell proliferation. Thus, HOTAIR functions as an oncogene and plays an important role in the pathogenesis of GC.

## 2. Materials and Methods

### 2.1. Patient Samples

54 GC tissue samples and matched adjacent normal tissues were obtained from patients who had undergone surgical resection in the Second Xiangya Hospital of Central South University (Changsha, China) and People's Hospital of Taizhou (Jiangsu, China), between January 2014 and December 2017. After surgical resection, tissues samples were immediately snap-frozen in liquid nitrogen and then stored in liquid nitrogen for further analysis. Tumor samples were diagnosed in accordance with World Health Organization (WHO) system, by two pathologists unaware of patient data. No radiotherapy or chemotherapy was administered before surgery. Written informed consent was collected from all patients. This study was approved by the Institutional Ethical Board of the Second Xiangya Hospital of Central South University (Changsha, China) and People's Hospital of Taizhou (Jiangsu, China)

### 2.2. Cell Culture

Four human GC cell lines AGS, MGC-803, SGC-7901, BGC-823, and a normal gastric epithelium cell line GES-1 were obtained from Center for Medical Research, the Second Xiangya Hospital, Central South University, China. Cells were cultured in RPMI -1640 (FBS, Gibco, Thermo Scientific, Waltham) supplemented with 10% fetal bovine serum (FBS, Gibco, Thermo Scientific). The cells were cultured in a humidified incubator at 37°C with 5% CO_2_.

### 2.3. Cell Transfection

The siRNA against HOTAIR or control siRNA was transfected into GC cells by using Lipofectamine 2000 according to the manufacturer's instructions. The siRNA against HOTAIR was purchased from GenePharma (Jiangsu, China).

### 2.4. RNA Extraction and qRT-PCR Assays

Total RNA was isolated from tissues or cells by using TRIZOL reagent (Invitrogen). Then, the RNA was reverse-transcribed to cDNA using a Reverse Transcription Kit (Promega Co., Madison, WI, USA). The mRNA levels were detected with a SYBR Premix Ex Taq (Takara, Dalian China). GAPDH was used as an endogenous control. All the Quantitative real-time PCR assays were performed on a Roche Detection System (Roche Applied Science). The qRT-PCR results were analyzed by a comparative threshold cycle (Ct) method and then converted into fold changes. All the experiments were performed at least for three times.

### 2.5. Cell Proliferation and Cell Cycle

Approximately 5 × 10^4^ GC cells were plated in 24-well plates and then transfected with miR-148a mimics or inhibitors or si-HOTAIR or negative control. After 24, 48. and 72 h, the cell numbers were detected by using a Z1 COULTER COUNTER Cell and Particle Counter (Beckman Coulter, Fullerton, CA, USA). For cell cycle analyses, the GC cells were seeded in 6-well plates and then transfected with si-HOTAIR. 48 h later, the transfection, cells were fixed in 70% ethanol at 4°C for 24 h and stained with propidium iodide (Beytime, Beijing, China). The cell cycle distribution was assessed by flow cytometry (BD FACS Calibur, American).

### 2.6. Western Blot Analysis

Cells were lysed using a 1× sodium dodecyl sulfate buffer. The protein concentrations were detected using a BCA Protein Assay kit. 30 *μ*g of proteins was separated by SDS-PAGE and transferred onto polyvinylidene fluoride membranes. The membranes were incubated with antibodies specific for P21, P53, and Bcl2 or GAPDH (Cell Signaling Technology) overnight at 4°C. Then, the blots were incubated with HRP-conjugated secondary antibodies for 2h and were detected by using enhanced chemiluminescence (Cell Signaling Technology).

### 2.7. Statistical Analysis

All statistical analysis was performed by using SPSS 21.0 and prism 11.0 software. The data are presented as the mean ± SD and determined by Student's t test. The relationships between HOTAIR expression and clinicopathological parameters were determined by chi-square test. And survival curves were estimated by the Kaplan-Meier method. P < 0.05 was considered statistically significant.

## 3. Results

### 3.1. HOTAIR Is Upregulated in GC Tissues and Cells

**Figure 1:**
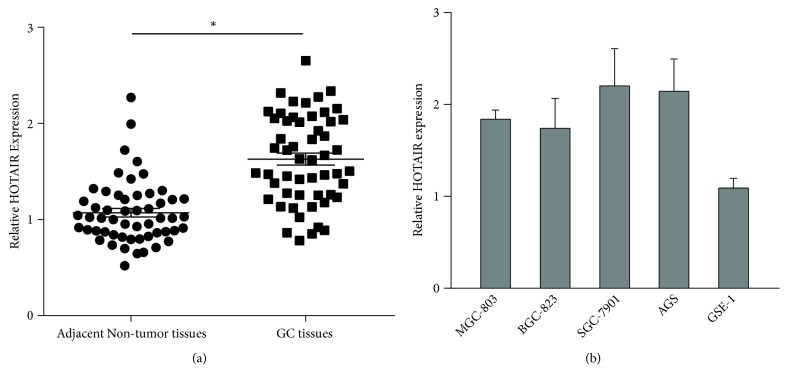
The expression levels of HOTAIR were significantly upregulated in GC tissues and cells. (a) Difference between the expression levels of HOTAIR in 54 pairs of GC tissues and adjacent nontumor tissues. (b) The expression levels of HOTAIR in human GC cell lines AGS, MGC-803, SGC-7901, BGC-823, and a normal gastric epithelium cell line GES-1. The expression levels of HOTAIR were normalized to GAPDH. *∗* P<0.05.

We firstly determined the expression levels of HOTAIR in GC tissues and adjacent noncancer tissues from 54 patients. The general and clinical characteristics of these patients were shown in Table 1. The expression level of one adjacent noncancer tissue was defined as 1. And we compared the expression level of HOTAIR in other tissues with it. As shown in Figure 1(a), the expression of HOTAIR in GC tissues was upregulated compared with adjacent noncancer tissues. Furthermore, we defined the expression level of HOTAIR > 1.40 (the average level of adjacent noncancer tissues + SD) as high expression. We analyzed the relationship between HOTAIR and clinicopathological parameters by using chi-square test. It was shown that HOTAIR expression was positively associated with tumor differentiation, lymph node and distant metastasis, and clinical stage (Table 1). We also analyzed the expression levels of HOTAIR with these parameters by using univariate analysis and multivariate analysis. The results also showed that there is a great difference of HOTAIR expression between groups with different tumor differentiation, lymph node, and distant metastasis. Then, we detected the expression levels of HOTAIR in four human GC cell lines (AGS, MGC-803, SGC-7901, BGC-823) and a normal gastric epithelium cell line (GES-1). The results showed that HOTAIR expression was higher in GC cells when compared with GES-1 cells (Figure 1(b)).

### 3.2. HOTAIR Is a Potential Diagnostic and Prognostic Marker

**Figure 2:**
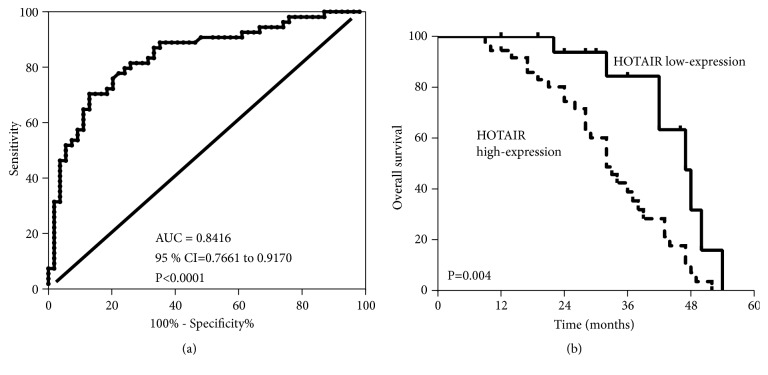
HOTAIR was a potential diagnostic and prognostic marker. (a) Receiver operation characteristics (ROC) curve for prediction of GC using HOTAIR expression level. (b) Survival of patients in HOTAIR low expression group and high expression group.

To determine the diagnostic value of HOTAIR in GC, we compared the difference between GC tissues and adjacent nontumor tissues from the ROC curves based on the cutoff (1.40). The area under the ROC curve (AUC) was up to 0.8416 (95 % CI=0.7661 to 0.9170, P<0.0001, Figure 2(a)). The sensitivity and specificity were 66.67 and 87.04%, respectively. The relationship between HOTAIR expression and patient overall survival was evaluated by Kaplan-Meier analysis. The results showed that higher HOTAIR expression was associated with a poor overall survival (p < 0.001, Figure 2(b)). The hazard ratio was 2.681, and 95% CI of ratio was 1.370 to 5.248. Collectively, these data indicated that HOTAIR is higher in GC tissues and cells and might be a potential diagnostic and prognostic biomarkers for GC.

### 3.3. HOTAIR Promotes GC Cell Proliferation

**Figure 3:**
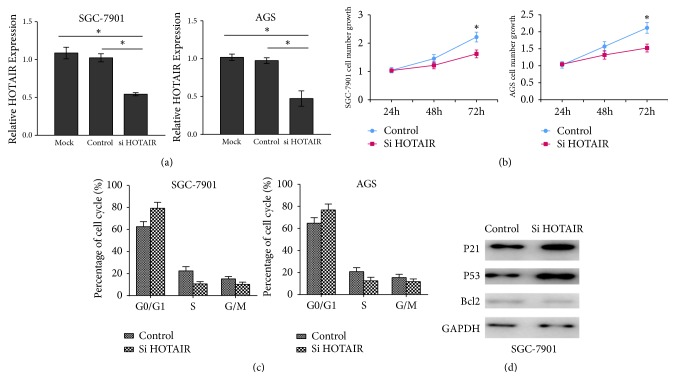
The effects of HOTAIR siRNA-transfection on GC cell proliferation. (a) The efficiency of HOTAIR siRNA transfection in SGC-7901 and AGS. (b) The effects of HOTAIR knockdown on SGC-7901 and AGS cell growth. (c) The effects of HOTAIR knockdown on cell cycle distribution in SGC-7901 and AGS cells. (d) The effects of HOTAIR knockdown on the protein levels of P21, P53, and Bcl2 in SGC-7901 cells.

To determine the effects of HOTAIR on GC cell proliferation, we employed HOTAIR siRNA to silence its expression in SGC-7901 and AGS (Figure 3(a)). The results indicated that the siRNA exhibited a higher knockdown efficiency of HOTAIR in SGC-7901 and AGS cells. And so, it was applied for HOTAIR silencing in the following experiments. Cell proliferation assays showed that knockdown of HOTAIR inhibited the cell proliferation rate in SGC-7901 and AGS cells (Figure 3(b)). Furthermore, cell cycle assays observed that inhibition of HOTAIR could alter cell cycle distribution. As shown in Figure 3(c), knockdown of HOTAIR induced G0-G1 cell cycle arrest, subsequently leading to a considerable decrease of cell percentage in the S-phase and a significant increase of cell percentage in G0/G1-phase in SGC-7901 and AGS cells. In addition, we detected the effect of HOTAIR on P21, P53, and Bcl2 protein expression and found that knockdown of HOTAIR could enhance the protein levels of P21 and P53 in SGC-7901 cells (Figure 3(d)). Thus, these data demonstrated that HOTAIR promotes GC cell proliferation.

## 4. Discussion

Gastric cancer (GC) is one most frequent causes of cancer-related deaths. Despite the advanced diagnostic and therapeutic techniques, the survival rate of those diagnosed with advanced GC remains unsatisfactory [1]. Therefore, better understanding of the pathogenesis of GC is essential for diagnosis and therapy.

Long noncoding RNAs (lncRNAs) are a group of noncoding RNA, which are greater than 200 nucleotides without evident protein coding function [1, 2]. Recently, lncRNAs have emerged as novel regulators in the initiation and progression of cancers. And the dysregulated lncRNAs can also act as potential diagnostic and prognostic biomarker for cancers [6, 10–12]. HOTAIR has been reported as an oncogene in several cancers [23–28]. Previous studies have shown that HOTAIR is elevated in lung cancer, breast cancer, liver cancer, and so on and correlates with metastasis and poor prognosis. Moreover, HOTAIR promotes proliferation, survival, invasion, metastasis, and drug resistance in the cancer cells [29–31]. In the present study, we found that HOTAIR was significantly upregulated in GC tissues and cells. In addition, higher HOTAIR expression was correlated with tumor differentiation, lymph node and distant metastasis, and clinical stage. Meanwhile, we compared the difference between GC tissues and adjacent nontumor tissues from the ROC. The sensitivity and specificity of HOTAIR for diagnosis were 66.67 and 87.04%, respectively. And the patients with higher HOTAIR expression had a worse survival. These findings indicated that upregulated HOTAIR may be involved in the tumorigenesis of GC, and it could be a good diagnostic and prognostic biomarker for GC.

lncRNAs can act as oncogene or tumor suppressor, contributing to caner carcinogenesis. For example, knockdown of HOTAIR can significantly suppress the cell proliferation of HepG2 cells and downregulate the protein expression levels of two proliferation markers Ki67 and proliferate cell nuclear antigen (PCNA). Furthermore, inhibition of HOTAIR induces G0/G1 cycle arrest by increasing p27 and decreasing cyclin D1 [32]. In our study, we demonstrated that HOTAIR could promote GC cell proliferation and alter the cell cycle distribution. Therefore, our data revealed that HOTAIR may act as an oncogene and promotes GC development.

In conclusion, we showed that HOTAIR is upregulated in GC and promotes cell proliferation. Thus, our findings revealed the involvement of HOTAIR in the pathogenesis of GC.

## Figures and Tables

**Table 1:** Association of HOTAIR expression with clinical and pathologic features in GC patients.

Parameter	Total	HOTAIR		*χ2*	P
		Low	High		
Age (years)				1.662	0.197
≤60	39(72.2%)	11(20.3%)	28(51.9%)		
>60	15(27.8%)	7(13.0%)	8(14.8%)		
Gender				0.000	1.000
Female	18(33.3%)	6(11.1%)	12(22.2%)		
Male	36(66.7%)	12(22.2%)	24(44.5%)		
Tumor size (cm)				0.947	0.331
<5	23(42.6%)	6(11.1%)	17(31.5%)		
≥5	31(57.4%)	12(22.2%)	19(35.2%)		
Tumor differentiation				11.910	0.001
Well/moderate	22(40.7%)	10(18.5%)	12(22.2%)		
Poor	32(59.3%)	8(14.8%)	24(44.4%)		
Lymph node metastasis				8.704	0.003
Negative	16(29.6%)	10(18.5%)	6(11.1%)		
Positive	38(70.4%)	8(14.8%)	30(55.6%)		
Distant metastasis				12.15	0.000
Negative	30(55.6%)	16(29.6%)	14(25.9%)		
Positive	24(44.4%)	2(3.7%)	22(40.7%)		
Clinical stage (TNM)				6.000	0.014
I~II	18(33.3%)	10(18.5%)	8(14.8%)		
III~IV	36(66.7%)	8(14.8%)	28(51.9%)		

## Data Availability

The data used to support the findings of this study are available from the corresponding author upon request.
